# Psychopathology and Alexithymia in Patients with Moderate-to-Severe Psoriasis: Development of a Novel Index with Prognostic Value

**DOI:** 10.3390/ijerph19074029

**Published:** 2022-03-29

**Authors:** Sofia Tsiori, Natalia Rompoti, Konstantinos Kontoangelos, Christos Papageorgiou, Charalabos Papageorgiou, Alexander Stratigos, Dimitrios Rigopoulos

**Affiliations:** 1 1st Department of Psychiatry, Eginition Hospital, Medical School, National & Kapodistrian University of Athens, 11528 Athens, Greece; kontoangel@med.uoa.gr (K.K.); chrispapageorgio@gmail.com (C.P.); chpapag@med.uoa.gr (C.P.); 2 1st Department of Dermatology-Venereology, Andreas Sygros Hospital, Medical School, National & Kapodistrian University of Athens, 16121 Athens, Greece; natalia.rompoti@gmail.com (N.R.); alstrat2@gmail.com (A.S.); dimitrisrigopoulos54@gmail.com (D.R.); 3 Neurosciences and Precision Medicine Research Institute “Costas Stefanis”, University Mental Health, 15601 Athens, Greece

**Keywords:** psoriasis, alexithymia, depression, mental illness, psychopathology

## Abstract

Background: Psoriasis is a chronic, relapsing, inflammatory disease with a high risk of developing mental health difficulties. Objective: The purposes of the study were to evaluate in moderate-to-severe psoriasis (a) the prevalence of depression and psychopathology, (b) the relationship between depression, psychopathology symptoms, and alexithymia, including its three dimensions, difficulty in identifying feelings (DIF), difficulty in describing feelings (DDF), and externally oriented thinking (EOT), and (c) to establish a novel index for the development of depression according to patients’ psychopathological profile. Methods: In 104 patients, alexithymia was evaluated with the Toronto Alexithymia Scale (TAS-20), depression with the Beck Depression Inventory (BDI), and psychopathology with the Brief Symptom Inventory SCL-90 (SCL90). A psychopathology index that combines information from the BDI and SCL90 scales was constructed and the performance of the index with alexithymia was examined. Results: Female patients and active smokers score higher on BDI and SCL90 scales. Overweight patients tend to score arithmetically higher. The psychopathology index developed correlates significantly with age, DIF, DDF, and TAS-20. DIF, DDF, and TAS-20 are significant predictors of the psychopathology index. Patients with alexithymia/possible alexithymia are six times as likely to score higher in one of the psychopathology scales. Conclusions: Alexithymia is a significant factor in the development of psychopathology in psoriasis patients. The use of the proposed novel psychopathology index could be essential in order to identify patients with moderate-to-severe psoriasis who are more likely to experience depression and psychopathology. This could have an impact on the decision-making of psoriasis treatment and monitoring of the patient.

## 1. Introduction

Psoriasis is a chronic, relapsing, inflammatory skin disease characterized by a variety of symptoms. It affects 2–4% of the general population in Western countries [1,2]. Psoriasis affects both sexes [3,4] and its pathogenesis is not fully understood. However, a number of risk factors are recognized, including genetic and environmental aspects [1,2,5].

Patients with psoriasis are more likely than patients with other dermatological conditions to have mental health difficulties [6,7,8,9]. The correlation of psoriasis with depression and anxiety disorders is confirmed [10,11,12] in numerous studies. Their appearance can trigger psoriasis but at the same time can hinder its treatment [2,13,14].

A personality trait studied in patients with psoriasis is alexithymia, invented by Sifneos to describe the difficulty of understanding, processing, and describing emotions [15]. Patients with alexithymia are characterized by limited imagination, externally oriented thinking, difficulty in separating emotions from the bodily senses, and a lack of empathy [16,17,18,19]. Freyberger introduced the concept of primary and secondary alexithymia [20]; the former is considered a genetic trait while the latter is considered a reaction to physical illness or a dramatic change in human life [21,22]. Alexithymia in this way can act as a triggering factor of psoriasis [23] or it can be the result of psoriasis, as this change can disrupt emotional communication and create alexithymia [19].

Past research has shown high rates of alexithymia in patients with moderate-to-severe psoriasis [24]. In addition, many studies have shown a positive association between alexithymia and depression [25,26,27,28] and other psychiatric disorders [28,29,30]. The purposes of the current study were (a) to investigate the prevalence of depression and psychopathology among patients with moderate-to-severe skin psoriasis, (b) to assess the relationship between depression, psychopathology symptoms, and alexithymia, as well as its three dimensions, difficulty in identifying feelings (DIF), difficulty in describing feelings (DDF), and externally oriented thinking (EOT), by taking into account demographic and clinical data of the patients. Moreover, an effort has been made to establish a novel index that can be useful in identifying who is at greater risk of developing a diagnosis of depression concerning patients with moderate-to-severe psoriasis. Its association with alexithymia and demographic characteristics of this study cohort were also investigated.

## 2. Materials and Methods

### 2.1. Sample and Procedure

The list of scheduled appointments of the dermatologic clinic was used in order to recruit 104 patients with confirmed diagnosis of moderate-to severe skin psoriasis with or without joint involvement. All patients were under classical and biological systemic therapies, and they participated in the study between April 2018 and March 2020 in Andreas Sygros Hospital of Cutaneous and Venereal Diseases. Toronto Alexithymia Scale (TAS-20), Beck Depression Inventory (BDI), and Brief Symptom Inventory SCL-90 (SCL-90) were administered to the psoriatic patients, while demographic and clinical data were collected as well. Patients with learning disability and illiteracy problems were excluded from this study.

### 2.2. Assessment Instruments

The 20-item self-report Toronto Alexithymia Scale (TAS-20): The scale includes 20 items rated on a 5-point Likert scale, ranging from 1 (strongly disagree) to 5 (strongly agree). The total score (TTL) of the scale ranges from 0 to 100. It consists of three subscales, measuring the difficulty in identifying feelings (DIF), the difficulty in describing feelings (DDF), and the externally oriented thinking (EOT). According to the accepted standard, a total score above 61 indicates alexithymia, a score between 52 and 60 suggests intermediate/borderline alexithymia, and a score lower than 51 indicates absence of alexithymia (30). In this paper, we use only two categories of TAS-20, using the cut-off point at 51 (TAS-20 binary). The TAS-20 has been translated and validated in Greek [31].

The Symptom Checklist-90 (SCL-90) [32]: It is a self-assessment questionnaire which measures nine psychopathology parameters: (1) somatization; (2) depression; (3) anxiety; (4) phobic anxiety; (5) obsessive compulsive; (6) paranoid ideation; (7) psychoticism; (8) hostility; (9) interpersonal sensitivity. The questionnaire includes 90 questions in total. All entries are rated from 0 to 4, giving a total score of 360. The scale is used to extrapolate three aggregate indexes: (a) the general severity index; (b) the positive symptoms distress index; (c) the positive symptoms total. A weighted Greek version is available. The General Symptomatic Index can be computed from the SCL-90 by simple arithmetic by adding all the raw factor scores and dividing by 90. The positive symptom distress level is the average level of distress of those symptoms out of 90, to which the patient indicates any degree of distress. The positive symptom total is a number of symptoms out of 90, to which the patient indicates any degree of distress [33].

The Beck Depression Inventory (BDI) is a 21-question multiple-choice self-assessment report inventory, one of the most widely used instruments for measuring the severity of depression. Its development marked a shift among health care professionals, who had until then viewed depression from a psychodynamic perspective. In its current version, the questionnaire is designed for individuals aged 13 and over and is composed of items related to symptoms of depression such as hopelessness and irritability, cognitions such as guilt or feelings of being punished, and as physical symptoms, such as fatigue, weight loss, and lack of interest in sex. Abbreviation has been stated, when the test is scored, that a value of 0 to 3 is assigned for each answer and then the total score is compared to a key to determine the depression severity. The standard cut-offs are as follows: 1–9 indicates minimal depression, 10–18 indicates mild depression, 19–29 indicates moderate depression, and 30–63 indicates severe depression [34].

### 2.3. Novel Psychopathology Index

A psychopathology index that combines information from the BDI and SCL90 scales was constructed. Even though depression subscale is included in SCL90 scale, we combined the two scales, as the BDI scale includes both cognitive and somatic symptoms of depression. Thus, assessment of the depression subscale can be more precise. Initially, the median value of the BDI scale and of each parameter in the SCL90 scale were calculated. For each scale, a score of 0 was given to a psoriatic patient when its value was below the median and 1 when the value was above or equal to the median. The proposed index is the sum of 10 binary scales (the binary versions of BDI and the nine dimensions of SCL90). The index ranges from 0 (when a patient scores below the median in all the scales) to 10 (when a patient scores higher than the median in all the scales), with higher values indicating higher psychopathology.

### 2.4. Statistical Analysis

A Kolmogorov–Smirnov test was performed to determine whether the psychopathology scales were normally distributed. The null hypotheses of the tests were rejected, and therefore the variables are presented through median and interquartile range. A Mann–Whitney test was used to examine the association of psychopathology scales and psychopathology index with the demographic and clinical characteristics. Spearman correlation coefficients were used to assess the relation of the psychopathology scales and index with age and the alexithymia scales.

The psychopathology index represents the number of “successes” in 10 questions (the binary versions of the psychopathology scales), where “success” is defined when a scale takes a value greater than the median. We assume that the psychopathology index follows a binomial distribution with 10 independent trials. Therefore, binomial regression analysis was performed to examine the association of the psychopathology index (dependent variable) with each of the alexithymia scales (independent variable). A quasibinomial distribution was used to handle the overdispersion problem. All models are controlled for gender and age. The statistical significance level was set at 0.05. Statistical analysis was performed using the Statistical Package for the Social Sciences (IBM SPSS Statistics for Windows, Version 24.0, accessed on University Mental Health, Neurosciences and Precision Medicine Research Institute “Costas Stefanis”, Athens, Greece) and the statistical program R (free software downloaded from http://www.r-project.org/, accessed on 23 March 2022) for performing the binomial regression analysis with quasibinomial distribution.

## 3. Results

Table 1 displays the descriptive statistics of the BDI, SCL90 scales, and psychopathology index (median and interquartile range) across the categories of gender, BMI (normal/overweight), current smoking status (yes/no), comorbidities (yes/no), concomitant PsA (yes/no), and TAS-20 (non-alexithymia/possible alexithymia or alexithymia). Among the psoriatic patients, females score higher on BDI and all the SCL90 scales than males. In particular, the median scores differ statistically significantly among males and females in BDI scale (6.0 vs. 11.0, *p* = 0.013), in somatization (4.0 vs. 9.0, *p* = 0.002), in interpersonal sensitivity (3.0 vs. 7.0, *p* = 0.033), in depression (5.0 vs. 13.0, *p* = 0.006), in anxiety (3.0 vs. 6.0, *p* = 0.031), and in phobic anxiety (1.0 vs. 2.0, *p* = 0.036). Besides that, males score lower in the psychopathology index than females (4.0 vs. 7.0, *p* = 0.014). Overweight psoriasis patients tend to score higher on the psychopathology scales than the patients with normal weight; however, the differences of the medians were not found to be statistically significant. Psoriatic patients who are currently smoking score higher on the BDI and SCL90 scales than the patients who are not smoking. The difference in the medians was not found to be statistically significant, except for the scale of phobic anxiety (2.0 vs. 0.0, *p* = 0.044). No significant differences between the scores of psoriatic patients with and without comorbidities, or with and without concomitant PsA, were found. Patients with possible alexithymia or alexithymia score statistically significant higher on BDI (16.5 vs. 5.0, *p* < 0.001), on somatization (12.0 vs. 3.0, *p* < 0.001), on obsessive-compulsive (16.5 vs. 5.0, *p* < 0.001), on interpersonal sensitivity (10.0 vs. 2.5, *p* < 0.001), on depression (19.0 vs. 3.5, *p* < 0.001), on anxiety (9.5 vs. 2.0, *p* < 0.001), on hostility (4.5 vs. 2.0, *p* < 0.001), on phobic anxiety (4.5 vs. 0.0, *p* < 0.001), on paranoid ideation (7.5 vs. 3.0, *p* < 0.001), on psychotism (6.0 vs. 1.0, *p* < 0.001), and on the psychopathology index (9.5 vs. 2.5, *p* < 0.001) than the patients with non-alexithymia.

Spearman correlation coefficients among the BDI, SCL90 scales, and psychopathology index with the variables of age and the scales of TAS-20 are given in Table 2. The scales of somatization, obsessive-compulsive, interpersonal sensitivity, depression, anxiety, hostility, phobic anxiety, and psychoticism are significantly correlated with the patients’ age with a negative correlation, implying that older patients score lower on the psychopathology scales. BDI and SCL90 scales correlate statistically significantly with the DIF, DDF, and the total score of TAS-20. Psoriatic patients with more difficulty identifying feelings or describing feelings score higher in the scales of BDI and SCL90. Besides that, patients with higher scores in the total score of alexithymia score higher in the scales of BDI and SCL90. The psychopathology index correlates significantly with age, DIF, DDF, and total score of TAS-20.

The binomial regression model was used to predict the psychopathology index for each scale of TAS-20 (Table 3). The scales of difficulty identifying feelings (DIF), of difficulty describing feelings (DDF), the total score of TAS-20, and its binary version are statistically significant predictors of the psychopathology index. One unit increase in the DIF or DDF scale corresponds to 20% or 21% increase in the probability that a patient will score higher than the median in one of the psychopathology scales (OR = 1.20 or OR = 1.21, *p* < 0.001). One unit increase in the total score of TAS-20 increases the probability of scoring higher than the median in one of the psychopathology scales by 8% (OR = 1.08, *p* < 0.001). Psoriatic patients diagnosed with possible alexithymia or alexithymia are six times more likely to score higher than the median in one of the psychopathology scales (OR = 5.99, *p* < 0.001) than patients without alexithymia.

## 4. Discussion

When it comes to assessing psychopathology, women with psoriasis appear to differ significantly from men in most parameters. Specifically, female patients presented with higher depression in BDI scale and higher somatization, interpersonal sensitivity, depression, anxiety, and phobic anxiety in SCL90 scale. Other studies have also shown that women with psoriasis are more likely to suffer from depression and anxiety than men [35,36]. Female patients tend to somatize more and experience higher phobic anxiety, which may be explained by the fact that women are less likely to accept their disease because it affects their appearance, resulting in a rise in negative emotions. Psoriatic patients often have feelings of shame and lack of confidence because of their disease. These emotions result in significant levels of life disruption as well as social withdrawal. The fear of rejection and criticism could explain higher rates of interpersonal sensitivity in women as they lead to feelings of personal inadequacy [37,38,39].

Overweight psoriasis patients tend to score higher on the psychopathology scales than the patients with normal weight. That can be explained by the fact that obesity makes psoriasis less susceptible to therapy, leading to disappointment each time a primary or secondary treatment failure occurs. Furthermore, obesity preserves a chronic systemic inflammation that also correlates with the severity of skin psoriasis [40]. Subsequently, obesity comes with a significant psychosocial burden, including issues related to mood, quality of life, and body image. Previous research suggests a relationship between excess body weight and depression [41].

Psoriatic patients who are currently smoking score higher on the BDI and SCL90 scales than the patients who are not smoking. In previous studies, smoking has not only been associated with disease onset, but also may contribute to the chronic nature of psoriasis in both sexes [42].

Patients with possible alexithymia or alexithymia score statistically significantly higher on depression, on all psychopathology subscales, and on the psychopathology index than patients with non-alexithymia. Alexithymia may be an expression of negative self-image, and difficulties in recognizing and expressing feelings can cause emotional numbness which is one of the core characteristics in depressive disorder [27]. Lack of emotional awareness can lead to limited social skills and feelings of personal inadequacy [17] which could increase levels of suspiciousness towards others [43]. In general, alexithymic patients’ inability to express their emotions can lead to difficulties in managing their internal stress [44]. In earlier studies, psychopathologists indicated that psychosis symptoms are related to disturbed emotional processes which can play a significant role in the development of cognitive-perceptual disturbances [45,46].

The scales of somatization, obsessive-compulsive, interpersonal sensitivity, depression, anxiety, hostility, phobic anxiety, and psychoticism are significantly correlated with the patients’ age with negative correlation, implying that older patients score lower on the psychopathology scales. Previous studies have shown that the development of better coping skills can be enhanced by age and maturity. In addition, acceptance of a chronic disease and the need for long-term treatment may be greater with age, with a further lower likelihood of societal stigmatization associated with facial disfigurement [47,48].

## 5. Novel Psychopathology Index

Depression and all psychopathology subscales were positively correlated with (1) difficulty describing feelings subscale, (2) difficulty identifying feelings subscale, and with (3) total alexithymia score. The above positive correlations are confirmed as the psychopathology index is correlated significantly with age, difficulty identifying feelings, difficulty describing feelings, and the total score of alexithymia.

Consequently, the scales of difficulty identifying feelings (DIF), difficulty describing feelings (DDF), and the total score of TAS-20 are statistically significant predictors of the psychopathology index. In general, psoriatic patients diagnosed with possible alexithymia or alexithymia are six times more likely to score higher than the median in one of the psychopathology scales than patients without alexithymia.

The contribution of this paper is the construction of a novel psychopathology index which measures psychopathology and depression more precisely. Even though depression subscale is included in the SCL90 scale, we combined the two scales, as BDI scale includes both cognitive and somatic symptoms of depression [49]. The SCL-90′s ability to discriminate patients from non-patients is adequate [50], but correlations with analogous and non-analogous measures have been controversial [51]. In particular, Rytilä-Manninen et al. (2016) suggested that the depression items in the SCL-90 measure general distress addressed by the whole questionnaire and that the depression scale does not reflect depression-specific factors of symptoms [52]. In contrast, the BDI scale evaluates key symptoms of depression including mood, pessimism, sense of failure, self-dissatisfaction, guilt, punishment, self-dislike, self-accusation, suicidal ideas, crying, irritability, social withdrawal, indecisiveness, body image change, work difficulty, insomnia, fatigability, loss of appetite, weight loss, somatic preoccupation, and loss of libido [53]. The BDI scale does not measure a nosological entity, but it measures the intensity of depression in accordance with the main symptoms of the depressive syndrome [54]. Depression is often underdiagnosed [55], so the psychopathology index will help us have a more precise picture of depression. A statistically significant positive association between the psychopathology index and alexithymia was identified. Psoriatic patients diagnosed with possible alexithymia or alexithymia are six times more likely to score higher than the median in one of the psychopathology scales (OR = 5.99, *p* < 0.001) than the patients without alexithymia. The use of this novel index could be essential for the clinician in order to identify patients with moderate-to-severe psoriasis who are more likely to experience depression and psychopathology. This could have an impact on both the treatment’s decision-making process with regards to psoriasis and the monitoring of the patient’s adherence and treatment’s success in the everyday practice, as these patients need psychological intervention in order to improve psychologically.

However, the proposed index serves as a starting point and requires further elaboration and development by specifying in which scales the patient exhibits values higher than the median.

The majority of studies regarding the relationship between alexithymia, depression, and anxiety conclude that they are strongly related [56,57]. Some studies have also indicated that increased prevalence of alexithymic features is found in patients with different mental disorders [58,59,60,61,62]. However, the assessment of psychopathology using the questionnaire Symptom Checklist-90-Revised (SCL-90-R), is mentioned in a few studies [29,63].

## 6. Limitations

Because of the COVID-19 pandemic, some scheduled appointments of the dermatologic clinic had to be cancelled, leading to disrupted collection of data and a smaller amount of eligible information than initially estimated.

## 7. Conclusions

Our research indicated that female patients presented with higher depression, somatization, interpersonal sensitivity, anxiety, and phobic anxiety. Furthermore, overweight psoriasis patients and those who are currently smoking tend to score higher on the psychopathology scales.

Since the discovery that patients with possible alexithymia or alexithymia score statistically significantly higher on depression, on all psychopathology subscales, and on the psychopathology index, the importance of alexithymia has made its appearance. Difficulty describing feelings subscale, difficulty identifying feelings subscale, and total alexithymia score were positively correlated with depression, psychopathology, and the psychopathology index. The aforementioned subscales and total score of alexithymia are statistically significant predictors of the psychopathology index. Consequently, alexithymia is a significant factor in the development of psychopathology in psoriasis patients. Clinicians should suspect the presence of alexithymic tendencies in psoriasis patients with psychopathology symptoms.

## Figures and Tables

**Table 1:** Median, interquartile range (IQR), and differences in the BDI and SCL90 scales by demographic and clinical characteristics.

	BDI	Somatization	Obsessive-Compulsive	Interpersonal Sensitivity	Depression	Anxiety	Hostility	Phobic Anxiety	Paranoid Ideation	Psychoticism	Psychopathology Index
	Median (IQR)										
Gender											
Male (*n* = 61)	6 (10.5)	4.0 (8.0)	6.0 (11.5)	3.0 (7.5)	5.0 (12.5)	3.0 (5.5)	2.0 (5.0)	1.0 (3.0)	3.0 (6.5)	1.0 (5.0)	4.0 (7.0)
Female (*n* = 43)	11.0 (12.0)	9.0 (13.0)	7.0 (15.0)	7.0 (9.0)	13.0 (14.0)	6.0 (9.0)	3.0 (6.0)	2.0 (6.0)	4.0 (9.0)	4.0 (8.0)	7.0 (6.0)
*p*	0.013	0.002	0.135	0.033	0.006	0.031	0.285	0.036	0.197	0.073	0.014
BMI											
Normal (*n* = 90)	8.5 (11.0)	5.0 (9.0)	7.0 (11.0)	4.0 (9.0)	8.5 (15.0)	4.0 (12.25)	2.0 (10.5)	1.0 (5.25)	3.0 (12.5)	2.0 (6.0)	5.0 (8.0)
Overweight (*n* = 14)	8.0 (10.75)	8.0 (20.0)	7.0 (17.25)	8.0 (14.0)	12.0 (17.0)	5.5 (6.0)	2.5 (4.0)	2.5 (7.25)	5.5 (6.0)	5.0 (8.5)	7.0 (9.0)
*p*	0.580	0.735	0.664	0.303	0.489	0.585	0.668	0.360	0.258	0.168	0.371
Smoker											
No (*n* = 45)	6.0 (13.0)	5.0 (9.5)	6.0 (7.0)	3.0 (7.0)	5.0 (15.0)	3.0 (7.0)	2.0 (5.0)	0.0 (3.0)	3.0 (4.0)	1.0 (5.0)	4.0 (7.5)
Yes (*n* = 57)	10.0 (10.5)	6.0 (11.0)	8.0 (12.5)	7.0 (9.5)	10.0 (14.5)	5.0 (10.0)	3.0 (5.5)	2.0 (5.0)	4.0 (8.0)	3.0 (6.5)	7.0 (7.5)
*p*	0.207	0.246	0.164	0.096	0.192	0.324	0.395	0.044	0.584	0.146	0.110
Comorbidities											
No (*n* = 45)	9.0 (10.5)	5.0 (10.5)	7.0 (12.5)	6.0 (11.5)	9.0 (14.5)	5.0 (7.5)	4.0 (5.0)	1.0 (4.0)	4.0 (7.5)	3.0 (6.5)	7.0 (9.5)
Yes (*n* = 56)	7.0 (11.75)	5.0 (8.5)	5.0 (13.75)	4.0 (7.75)	8.0 (15.0)	3.0 (9.0)	2.0 (4.0)	1.0 (4.5)	3.0 (7.75)	2.0 (5.0)	4.0 (7.75)
*p*	0.94	0.686	0.596	0.411	0.962	0.484	0.32	0.813	0.361	0.306	0.429
Concomitant PsA											
No (*n* = 74)	7.5 (10.5)	5.0 (10.0)	7.0 (13.5)	4.0 (9.0)	9.5 (15.25)	4.0 (9.0)	2.0 (6.0)	1.0 (5.0)	3.5 (8.0)	2.0 (6.0)	5.0 (8.0)
Yes (*n* = 29)	10.0 (13.5)	8.0 (15.0)	6.0 (9.5)	5.0 (9.5)	7.0 (15.0)	4.0 (8.5)	3.0 (6.0)	1.0 (3.0)	4.0 (6.5)	4.0 (5.5)	8.0 (8.0)
*p*	0.307	0.074	0.906	0.468	0.956	0.982	0.438	0.641	0.206	0.286	0.488
TAS-20											
non-alexithymia (*n* = 68)	5.0 (8.75)	3.0 (6.5)	4.5 (8.0)	2.5 (7.0)	3.5 (10.75)	2.0 (4.75)	2.0 (4.0)	0.0 (2.0)	3.0 (5.0)	1.0 (4.0)	2.5 (7.75)
possible alexithymia or alexithymia (*n* = 36)	16.5 (13.0)	12.0 (14.75)	16.0 (14.0)	10.0 (13.5)	19.0 (16.0)	9.5 (11.75)	4.5 (8.5)	4.5 (7.5)	7.5 (10.75)	6.0 (9.75)	9.5 (4.0)
*p*	<0.001	<0.001	<0.001	<0.001	<0.001	<0.001	<0.001	<0.001	<0.001	<0.001	<0.001

Abbreviations: BDI, Beck Depression Inventory; BMI, body mass index; IQR, interquartile range; PsA, psoriatic arthritis; TAS-20, Toronto Alexithymia Scale-20.

**Table 2:** Spearman correlation coefficients of BDI and SCL90 scales with the TAS-20 scales.

	BDI	Somatization	Obsessive-Compulsive	Interpersonal Sensitivity	Depression	Anxiety	Hostility	Phobic Anxiety	Paranoid Ideation	Psychoticism	Psychopathology Index
Age (y)	−0.163	−0.213 *	−0.248 *	−0.341 **	−0.218 *	−0.261 **	−0.291 **	−0.197 *	−0.171	−0.205 *	−0.239 *
DIF	0.653 **	0.650 **	0.651 **	0.648 **	0.702 **	0.667 **	0.529 **	0.526 **	0.513 **	0.557 **	0.685 **
DDF	0.545 **	0.472 **	0.541 **	0.556 **	0.568 **	0.505 **	0.407 **	0.430 **	0.388 **	0.444 **	0.525 **
EOT	0.104	0.106	0.024	0.015	0.066	0.089	−0.011	0.130	−0.032	−0.021	0.035
TTL	0.580 **	0.562 **	0.535 **	0.547 **	0.602 **	0.574 **	0.423 **	0.477 **	0.377 **	0.454 **	0.563 **

** *p* < 0.01, * *p* < 0.05. Abbreviations: BDI, Beck Depression Inventory; DDF, difficulty describing feelings; DIF, difficulty identifying feelings; EOT, externally oriented thinking; SCL90, Brief Symptom Inventory SCL-90; TAS-20, Toronto Alexithymia Scale-20; TTL, total score of TAS-20 scales; y, years.

**Table 3:** Binomial regression analyses for the prediction of the psychopathology index by each scale of TAS-20.

		OR	95 CI of OR	*p*
Psychopathology index	DIF	1.20	1.14–1.27	<0.001
	DDF	1.21	1.13–1.29	<0.001
	EOT	1.08	0.93–1.07	0.982
	TTL	1.08	1.05–1.11	<0.001
	TAS-20 binary	5.99	3.00–12.62	<0.001
Models are controlled for age and gender				

Abbreviations: DDF, difficulty describing feelings; DIF, difficulty identifying feelings; EOT, externally oriented thinking; OR, odd ration; TAS-20, Toronto Alexithymia Scale-20; TTL, total score of TAS-20 scales.

## Data Availability

All data can be found on 1st Department of Dermatology-Venereology, Andreas Sygros Hospital, Medical School, National and Kapodistrian University of Athens.
